# Double-End Location Technology of Partial Discharge in Cables Based on Frequency-Domain Reflectometry

**DOI:** 10.3390/s25154710

**Published:** 2025-07-30

**Authors:** Wang Miao, Hongjing Liu, Ci Song, Hongda Li, Nan He, Jingzhu Teng, Baoqin Cao, Ruonan Bai, Xianglong Li, Haibao Mu

**Affiliations:** 1 State Grid Beijing Electric Power Research Institute, Beijing 100075, China; miaowang402@163.com (W.M.); 15201137896@163.com (H.L.); a116152678@163.com (H.L.); 15021066716@163.com (N.H.); tengjingzhu@sina.com (J.T.); 15235123167@163.com (B.C.); 18002129795@163.com (R.B.); 15901212419@163.com (X.L.); 2 State Key Laboratory of Electrical Insulation and Power Equipment, Xi’an Jiaotong University, Xi’an 710049, China; songci@stu.xjtu.edu.cn

**Keywords:** cable partial discharge, double-ended measurement, frequency-domain reflectometry, region determination, location method

## Abstract

To realize the region determination and accurate location of cable partial discharge, this paper proposes a cable partial discharge double-end location technique based on frequency-domain reflectometry. The cable partial discharge double-end location technique based on frequency-domain reflectometry mainly includes the frequency band modulation technique and partial discharge location method. The frequency band modulation technique determines the effective frequency band range of the acquired cable transfer function through the frequency band range of the partial discharge signals measured at both ends, which ensures the reliability of the transfer function. The partial discharge location method constructs the cable partial discharge location function and the region determination function via spectral analysis of the cable transfer function obtained from the partial discharge signals, which realizes region determination and determines precise location of the cable partial discharge, respectively. Our simulation and experiment show that the cable partial discharge double-end location technique based on frequency-domain reflectometry can effectively determine the existence region of cable partial discharge and its accurate location (with a location error of less than 1%), showing good potential for practical application in engineering.

## 1. Introduction

With the development of industrialization and urbanization, power cables have gradually become the main carriers of electric energy transmission [1,2,3,4,5,6]. However, with the increase in operating years, some local defects will appear in the cable under the influence of operating conditions and external environment. The early detection of defects helps to ensure the safe and stable operation of the power transmission system. Partial discharge is one of the typical characteristics of local defects in cables under power frequency excitation [7,8,9,10,11]. The location of defects through partial discharge signal characteristics has significant engineering application value as it helps to detect defects in time and avoid defects from being transformed into permanent faults.

The methods currently used in cable defect partial discharge technology are mainly the threshold method, peak method, energy method, and correlation method. The threshold method [12] is mainly used to obtain the onset time of the partial discharge signal via the setting value, which is simple in principle but has a large error and poor anti-interference ability. The peak method [13], on the other hand, determines the signal delay from the peak of the discharge signal. In the propagation process, the dispersion and attenuation effects [14] will lead to the distortion of the partial discharge signal waveform. Therefore, obtaining the delay of the partial discharge signal only through the peak value will result in a large error. The energy method [15,16] determines the time delay by calculating the cumulative energy inflection point of the partial discharge signal. This method has strong noise immunity, but the inflection point selection method lacks stability. The correlation method is categorized into the direct correlation method and the generalized correlation method. The direct correlation method [17,18] calculates the similarity of the partial discharge signal through the mutual correlation algorithm to determine the signal delay. This method possesses strong noise immunity but is also limited by the dispersion and attenuation effects during the propagation of the partial discharge signal. The generalized correlation method [19,20,21] determines the time delay through the mutual power spectrum of the partial discharge signal, which overcomes the drawbacks of the direct correlation method (which is limited by the attenuation effect) and can effectively improve the location precision. However, this method has not yet provided a clear approach for determining whether the partial discharge signal is inside or outside the detected cable.

In view of the problems arising from the traditional partial discharge location techniques, this paper proposes a double-ended location technique for partial discharge in cables based on frequency-domain reflectometry. Frequency-domain reflectometry is currently widely used in cable defect detection based on the active injection of a signal. Herein, this method is used for the first time for the partial discharge location of defects in cables. The cable partial discharge double-end location technique based on frequency-domain reflectometry mainly includes the frequency band modulation technique and partial discharge location method. The frequency band modulation technique is used to determine the effective frequency band range for obtaining the cable transfer function through the partial discharge signal. The partial discharge location method distinguishes whether the partial discharge signal is within the cable from the polarity and can accurately locate the partial discharge defect. The cable partial discharge double-end location technique based on frequency-domain reflectometry proposed in this paper is successfully verified in a simulation and experiment.

## 2. Principle of Partial Discharge Double-End Location via Frequency-Domain Reflectometry

### 2.1. Basic Principles

**Figure 1:**
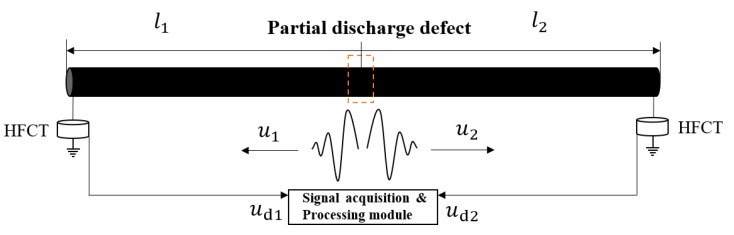
Schematic diagram of partial discharge propagation in cable.

A partial discharge propagation schematic of a cable partial discharge defect generated under power frequency excitation is shown in Figure 1. When the partial discharge defect generates the discharge signal, the partial discharge signal propagates to the two ends of the cable, respectively. The discharge signal transmitted to the first and last ends of the cable can be captured by high-frequency current sensors configured on the ground line of the cable [22]. The frequency-domain expressions of partial discharge signals collected at the first and last ends of the cable are as follows:

(1) $$U_{d1}=U_{1}e^{-\gamma l_{1}}\Gamma_{d1}$$

(2) $$U_{d2}=U_{2}e^{-\gamma l_{2}}\Gamma_{d2}$$

where $U_{1}$ is the frequency-domain expression of the initial partial discharge signal transmitted to the first end of the cable; $U_{2}$ is the frequency-domain expression of the initial partial discharge signal transmitted to the last end of the cable;*γ = α + jβ* is the cable propagation coefficient; α is the attenuation coefficient; β is the phase constant; $l_{1}$ is the length of the discharge defect from the first end of the cable; $l_{2}$ is the length of the discharge defect from the last end of the cable; $\Gamma_{d1}$ is the measurement factor for the first end of the cable; and $\Gamma_{d2}$ is the measurement factor for the last end of the cable.

Typically, the initial partial discharge signals transmitted to the first and last ends of the cable are consistent, $U_{1}=U_{2}$, and the same high-frequency current sensors are used, $\Gamma_{d1}=\Gamma_{d2}$. Thus, the transfer function of the cable can be obtained from the partial discharge signals measured at both ends of the cable:

(3) $$\Gamma_{pd}=\frac{U_{d2}}{U_{d1}}=e^{-\gamma {(l}_{2}-l_{1})}$$

In Equation (3), it is shown that the defect location information is contained in the transfer function obtained from the partial discharge signals measured at both ends of the cable.

### 2.2. Cable Transfer Function Acquisition Method Based on Partial Discharge Signals

From Section 2.1, it can be seen that the cable transfer function is the key to locating the discharge defects in the cable. The transfer function containing the information of the discharge defect can be obtained using the frequency-domain expression of the local discharge signal measured at the first and last ends of the cable. The frequency-domain expressions of the partial discharge signals at both ends of the cable can be obtained using the discrete Fourier transform of the time-domain signals:

(4) $$u_{d10}=\left[u_{d1},\;0\right]$$

(5) $${\tilde{u}}_{d10}=H\left(u_{d10}\right)$$

(6) $$U_{d1}\left[k\right]=\sum_{n=1}^{N}{\tilde{u}}_{d10}\left[n\right]\cdot e^{\frac{-j2\pi \left(k-1\right)\left(n-1\right)}{N}}\;\;\;\;\;(1\leq k\leq N)$$

(7) $$u_{d20}=\left[u_{d2},\;0\right]$$

(8) $${\tilde{u}}_{d20}=H\left(u_{d20}\right)$$

(9) $$U_{d2}\left[k\right]=\sum_{n=1}^{N}{\tilde{u}}_{d20}\left[n\right]\cdot e^{\frac{-j2\pi \left(k-1\right)\left(n-1\right)}{N}}\;\;\;\;\;\;(1\leq k\leq N)$$

(10) $$f\left[k\right]=\frac{\left(k-1\right)\cdot T_{s}}{N}\;\;\;\;\;\;\;\;\;(1\leq k\leq N)$$

where *$u_{d1}$* and $u_{d2}$ are the time-domain detection signals measured by two sensors at the first and last ends of the cable, respectively; $u_{d10}$ and $u_{d20}$ are the time-domain detection signals after zero-completion, respectively; H is the Hilbert transform; ${\tilde{u}}_{d10}$ and ${\tilde{u}}_{d20}$ are the complex signals after the Hilbert transform of $u_{d10}$ and $u_{d20}$, respectively; k is the frequency-domain sequence index; n is the time-domain sequence index; f is the frequency; N is the number of sequences; and $T_{s}$ is the sampling rate.

From Equation (3), the cable transfer function can be obtained by dividing Equations (6) and (9). It is known from Equation (10) that the frequency band range depends on the sampling rate of the signal. Although the partial discharge signal is usually a narrow-band signal concentrated in MHz, there is also an energy distribution in the high-frequency part. The cable transfer function obtained directly using Equations (6) and (9) will have serious interference with the discharge defect location result because of the low energy distribution in the high-frequency part.

Therefore, how to choose the frequency band range of the acquired cable transfer function is the key to ensuring the accuracy of the discharge defect location result. To solve this problem, this paper proposes the frequency band modulation technique. The specific steps of the frequency band modulation technique are as follows:

1. Take the maximum value of the frequency-domain expression, $U_{d1}$, of the partial discharge signal obtained at the first end of the cable and record its corresponding frequency.
2. Take the two frequency points corresponding to 5% of the maximum value of $U_{d1}$ as the effective frequency band range of the first-end partial discharge signal. The two frequency points are located to the left and right of the maximum frequency point, respectively, and ensure that the effective frequency band is minimized.
3. Take the maximum value of the frequency-domain expression, $U_{d2}$, of the partial discharge signal obtained at the last end of the cable and record its corresponding frequency.
4. Take the two frequency points corresponding to 5% of the maximum value of $U_{d2}$ as the effective frequency band range of the second-end partial discharge signal. The two frequency points are located to the left and right of the maximum frequency point, respectively, and ensure that the effective frequency band is minimized.
5. Take the intersection of the effective frequency bands of the first and second partial discharge signals as the effective frequency band of the cable transfer function.

According to the frequency band modulation technique, the cable transfer function obtained by the partial discharge signals is as follows:

(11) $$\Gamma_{pd}[k]=\frac{U_{d2}\left[k\right]}{U_{d1}\left[k\right]}\;\;\;\;\;\;\;\;\;\;\;\;\;\;\;\;\;\;\;\;(N_{1}\leq k\leq N_{2})$$

where $N_{1}$ and $N_{2}$ are the indexes of the cable transfer function obtained according to the frequency band modulation technique, respectively.

### 2.3. Location Method Based on Frequency-Domain Reflectometry

After obtaining the cable transfer function, the cable diagnostic function can be obtained via a spectral analysis of $\Gamma_{pd}$:

(12) $$D\left(x\right)=\sum_{k=N_{1}}^{N_{2}}\Gamma_{pd}\left[k\right]\cdot C\left[k\right]\cdot e^{j\beta (k)x}\cdot \Delta f\;\;\;\;\;(0\leq x\leq l)$$

where x is the length from the first end of the cable; C is the Chebyshev window function; Δf is the frequency interval; and l is the full length of the cable. Based on the cable diagnostic function, the cable partial discharge location function and region determination function can be obtained:

(13) $$D_{loc}\left(x\right)=\left|D\left(x\right)\right|\;$$

(14) $$D_{ch}(x)=real(D\left(x\right))$$

where $D_{loc}\left(x\right)$ is the cable partial discharge location function, obtained by taking the modulus of $D\left(x\right)$, and $D_{ch}(x)$ is the region determination function, obtained by taking the real part of $D\left(x\right)$. By default, the reference direction of the high-frequency current sensors at the first and last ends point to the monitored cable center, and the specific steps for locating partial discharges in cables are as follows:

1. Take the maximum value of the cable partial discharge location function $D_{loc}\left(x\right)$ as the initial location result of the partial discharge.
2. Input the initial location result into the region determination function.
3. If the value of the region determination function is positive, it is determined that the partial discharge signal originates from the inside of the monitored cable. The location of the partial discharge can be obtained by combining the total length of the cable, $l=l_{2}+l_{1}$, and the initial location result, $l_{2}-l_{1}$.
4. If the value of the region determination function is negative, it is determined that the partial discharge signal originates from the outside of the monitored cable. The initial location result is the distance between the two high-frequency current sensors.

**Figure 2:**
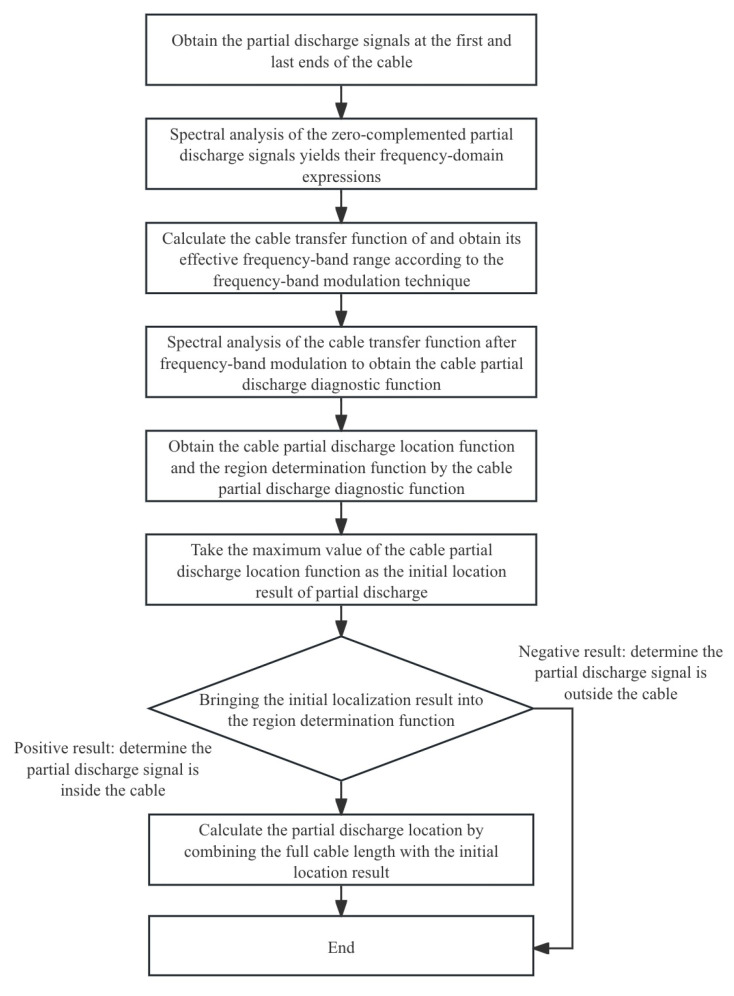
Flowchart of the partial discharge double-end location technique based on frequency-domain reflectometry.

Figure 2 shows a flowchart of the partial discharge double-end location technique based on frequency-domain reflectometry.

## 3. Simulation

In order to verify the reliability of the cable partial discharge double-end location technique based on frequency-domain reflectometry, a partial discharge signal transmission model for a 10 kV distribution cable was constructed in ADS. Situations where partial discharges occur inside and outside the cable were simulated, and the diagnostic results are given below.

**Figure 3:**
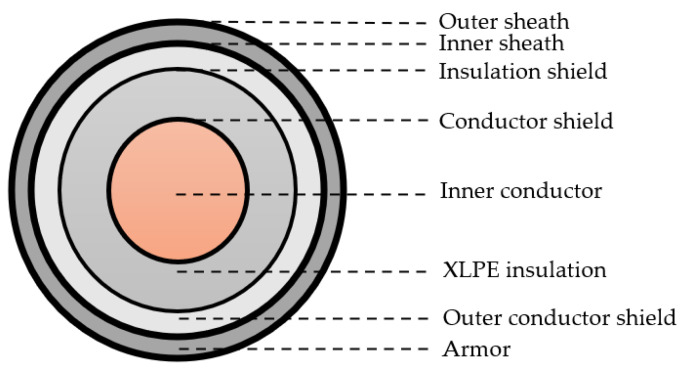
Schematic structure of 10 kV distribution cable.

Figure 3 shows the schematic structure of the 10 kV distribution cable, and the main parameters are shown in Table 1. In the ADS simulation, the signal sampling rate was set to 250 MHz. The partial discharge signals were simulated using a double exponential decay oscillator function with a center frequency of 5 MHz.

### 3.1. Partial Discharge Inside the Cable

**Figure 4:**
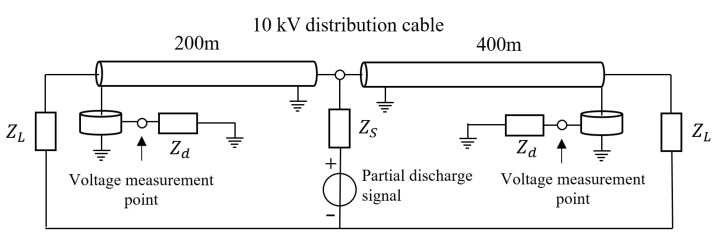
Partial discharge signal transmission model for 10 kV distribution cable constructed in ADS (intra-regional partial discharge).

Figure 4 shows the model of partial discharge signal transmission within the 10 kV cable constructed in ADS. The cable length was 600 m. The location of the discharge defect was set to 200 m from the first end of the cable, and the partial discharge signal of the outer conductor was measured with a high-frequency current transformer, which was configured at the first and last ends of the cable with the reference direction pointing to the center of the monitored cable.

**Figure 5:**
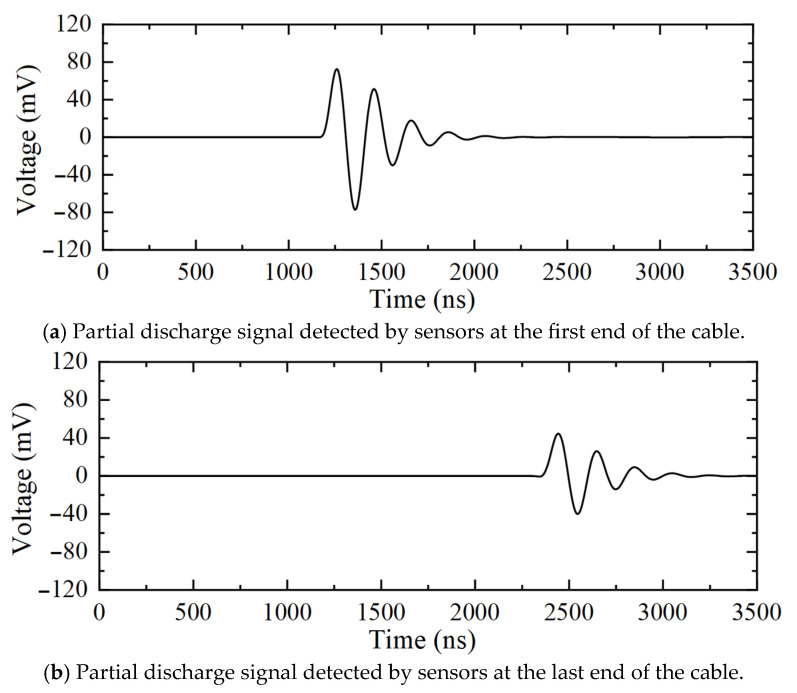
Time-domain signals for partial discharge detection in the 10 kV distribution cable (intra-regional partial discharge).

**Figure 6:**
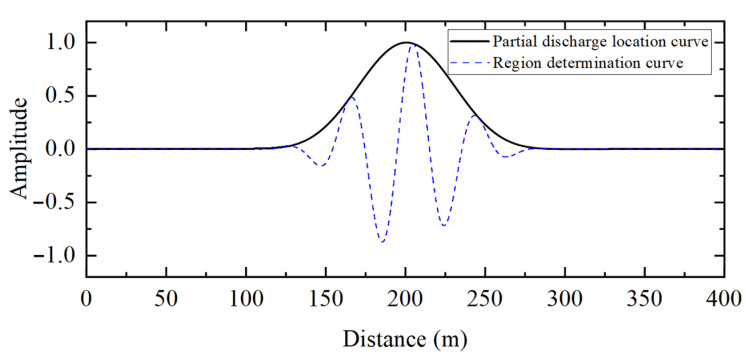
Partial discharge detection results of the 10 kV distribution cable based on frequency-domain reflectometry (intra-regional partial discharge).

Figure 5 shows the partial discharge signals detected by the sensors at the first and last ends of the cable when partial discharge occurs in the 10 kV distribution cable. Figure 6 shows the detection results of the frequency-domain reflectometry technique proposed in this paper for partial discharge signals. The peak value of the partial discharge location curve in Figure 6 is 200.60 m, and the corresponding region’s determination curve at this location is positive, which indicates that the partial discharge exists in the cable. Combining the full length of the cable yields a partial discharge location of 199.70 m from the first end.

Table 2 shows a comparison of the results of different methods for the location of the partial discharge. It is not difficult to conclude the following: not only can the method proposed in this paper determine whether the partial discharge signal exists inside the cable, but it can also locate it with much smaller than other methods. This is essentially because frequency-domain reflectometry takes into account the dispersion and attenuation effects of the partial discharge transmission process, thus ensuring a more accurate location for the discharge defect. In addition, the location accuracy of the generalized correlation method may also be high. However, no algorithm for partial discharge area determination has been developed for the generalized correlation method.

### 3.2. Partial Discharge Outside the Cable

**Figure 7:**
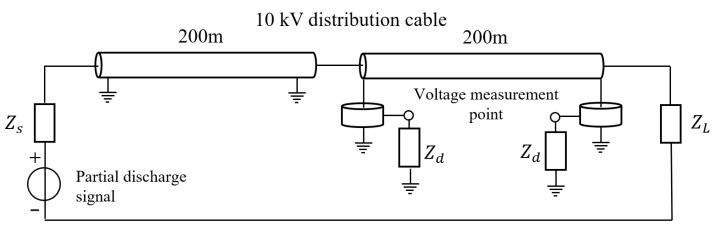
Partial discharge signal transmission model for 10 kV distribution cable constructed in ADS (inter-regional partial discharge).

As outlined in this section, to verify the reliability of the algorithm in cases where the partial discharge occurs outside the cable, we constructed a model of partial discharge signal transmission outside the 10 kV distribution cable in ADS. The model structure in ADS is shown in Figure 7. The cable length was 200 m and the partial discharge signal was 200 m from the first end of the cable.

**Figure 8:**
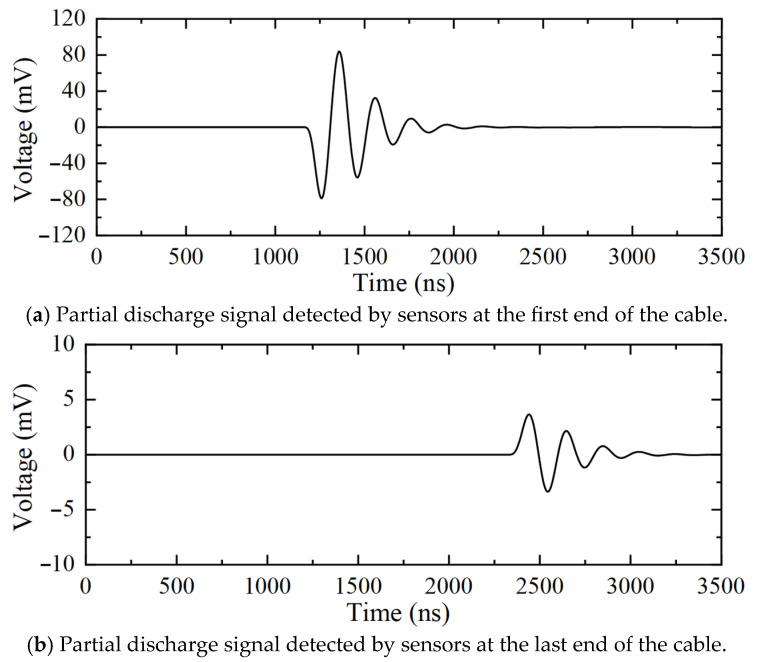
Time-domain signals for partial discharge detection in the 10 kV distribution cable (inter-regional partial discharge).

**Figure 9:**
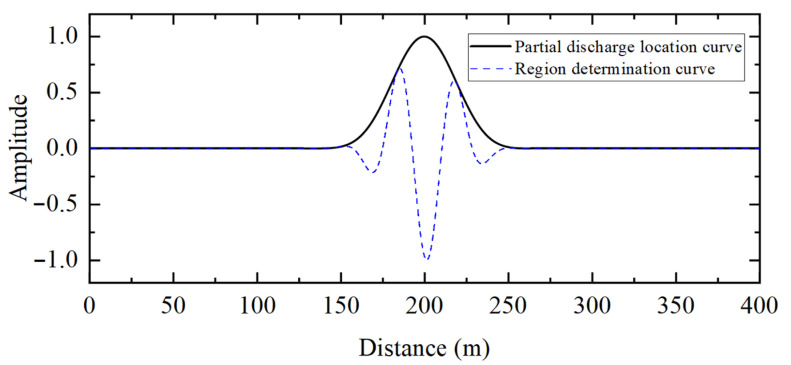
Partial discharge detection results of 10 kV distribution cable based on frequency-domain reflectometry (inter-regional partial discharge).

Figure 8 shows the partial discharge signals detected by the sensors at the first and last ends of the cable when the partial discharge occurs outside the 10 kV distribution cable. Figure 9 shows the detection result of the frequency-domain reflectometry method proposed in this paper. The peak value of the partial discharge location curve in Figure 9 is 200.31 m. However, the corresponding region determination curve for this location is negative, indicating that the partial discharge exists outside the cable. Thus, the peak of the location curve indicates the distance between the two sensors configured at the first and last ends of the cable. The above detection results show that the algorithm proposed in this paper can effectively determine whether the partial discharge is occurring outside the cable.

## 4. Experiment

**Figure 10:**
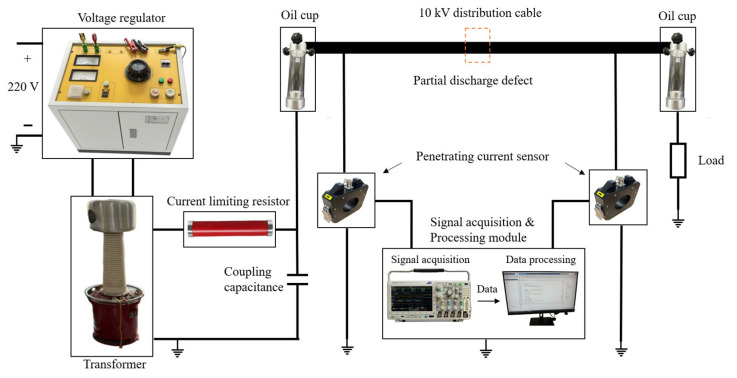
The experimental platform.

In order to verify the effectiveness of the frequency-domain reflection method proposed in this paper for the location of partial discharges in engineering practice, relevant experimental studies were carried out, as outlined in this section. The experimental platform built in this study is shown in Figure 10.

As shown in Figure 10, the object of the experiment was a 10 kV distribution cable with a length of 150 m and a discharge defect 50 m from the first end. Applying high-voltage power frequency excitation to the 10 kV distribution cable using a voltage regulator and transformer led to a partial discharge in the defect. The synchronized detection of partial discharge signals at the first and last ends of the cable was carried out using high-frequency current transformers with a bandwidth ranging from 500 kHz to 30 MHz. When the actual detected partial discharge signal noise is too large, it should be denoised before using the algorithm for location. Partial discharge-denoising algorithms have been reported extensively in related studies [23,24,25,26] and will not be repeated.

**Figure 11:**
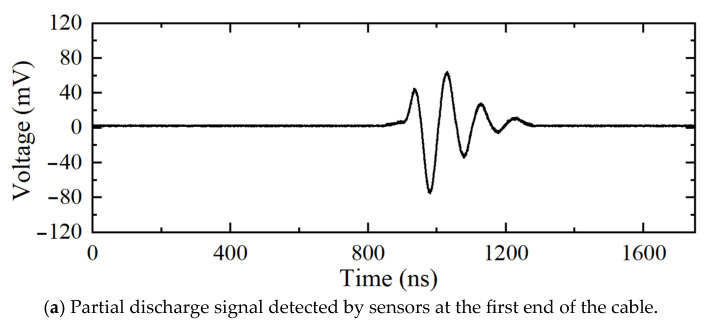

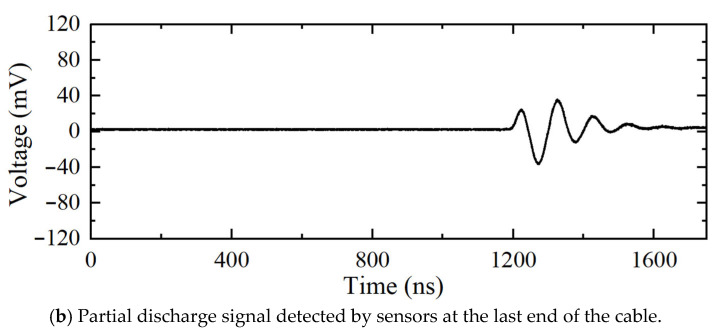
Partial discharge measured in the 10 kV distribution cable.

**Figure 12:**
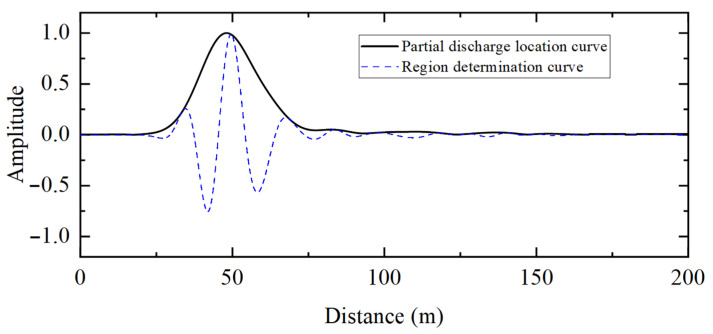
Location results of measured partial discharges in the 10 kV distribution cable based on frequency-domain reflectometry.

Figure 11 shows the measured partial discharge signals at the first and last end of the 10 kV distribution cable. Figure 12 shows the location results for the partial discharge using frequency-domain reflectometry.

The peak position of the partial discharge location curve is 48.08 m, and the corresponding value of the region determination curve is positive. Thus, it can be determined that the discharge defect exists inside the cable. Considering with the full cable length, it can be calculated that the discharge defect is 50.96 m from the first end of the cable, which is an error of less than 1 m from the real cable discharge defect location of 50 m.

It can be seen that the cable partial discharge double-end location technology based on frequency-domain reflectometry proposed in this paper could accurately detect the partial discharge location in the experiment, and so it has significant value for engineering applications.

## 5. Conclusions

This paper proposes a cable partial discharge double-end location technique based on frequency-domain reflectometry, which mainly includes the frequency band modulation technique and a partial discharge signal location algorithm, aiming to meet the needs of region determination and accurate location of cable partial discharge. Our simulation and experiment verify the reliability of the algorithm proposed in this paper, and the specific conclusions are as follows:

1. In order to ensure the reliability of the cable transfer function obtained by using the partial discharge signals measured at both ends of the cable, the frequency band modulation technique is proposed. The frequency band modulation technique obtains the effective frequency band range of the transfer function according to the frequency band range of the partial discharge signals measured at both ends of the cable, which ensures the reliability of the transfer function.
2. In order to achieve the region determination and obtain the accurate location of the partial discharge in a cable, the partial discharge location method based on frequency-domain reflectometry is proposed. The partial discharge location method constructs the cable partial discharge location function and the region determination function through a spectral analysis of the cable transfer function. Using the partial discharge location function peaks and region determination function symbol, one can effectively determine whether the partial discharge exists inside the cable and locate it accurately.
3. The simulation and experiment show that the cable partial discharge double-end location technology based on frequency-domain reflectometry proposed in this paper can effectively overcome the shortcomings of the traditional partial discharge location algorithm due to the dispersion and attenuation effect resulting in a large location deviation, and it can also realize the region determination and accurate location of the discharge defect.

## Figures and Tables

**Table 1:** Structural and electrical parameters of 10 kV distribution cable.

Type	Title	Value
Structural parameters	Inner conductor radius (mm)	3.51
	Conductor shield thickness (mm)	0.38
	XLPE insulation thickness (mm)	5.04
	Insulation shield thickness (mm)	0.38
	Outer conductor shield radius (mm)	9.31
Electrical parameters	Inner conductor conductivity (S/m)	5.71 × 10^7^
	Outer conductor conductivity (S/m)	5.71 × 10^7^
	XLPE insulation relative dielectric constant	2.3
	Relative permeability	1

**Table 2:** Comparison of different methods for localizing partial discharge.

Method	Location (m)	Location Error (%)	Region Determination
Peak method [13]	196.03	1.99	×
Energy method [15]	196.84	1.58	×
Direct correlation method [18]	197.56	1.22	×
Frequency-domain reflectometry	199.70	0.15	√

## Data Availability

Data are contained within the article.

## References

[1] Zhou C., Yi H., Dong X. (2017). Review of recent research towards power cable life cycle management. High Volt..

[2] Nadolny Z. (2022). Electric field distribution and dielectric losses in XLPE insulation and semiconductor screens of high-voltage cables. Energies.

[3] Zhou K., Tang Z., Meng P., Huang J., Xu Y., Rao X. (2023). A novel method for sign judgment of defects based on phase correction in power cable. IEEE Trans. Instrum. Meas..

[4] Shadi M.R., Mirshekali H., Shaker H.R. (2025). Partial Discharge-Based Cable Vulnerability Ranking with Fuzzy and FAHP Models: Application in a Danish Distribution Network. Sensors.

[5] Bakhshizadeh M.K., Vilmann B., Kocewiak Ł. (2022). Modal aggregation technique to check the accuracy of the model reduction of array cable systems in offshore wind farms. Energies.

[6] Zhang W., Song Y., Wu X., Liu H., Tian H., Tang Z., Xu S., Chen W. (2025). Detecting Partial Discharge in Cable Joints Based on Implanting Optical Fiber Using MZ–Sagnac Interferometry. Sensors.

[7] Montanari G.C. (2016). Partial discharge detection in medium voltage and high voltage cables: Maximum distance for detection, length of cable, and some answers. IEEE Electr. Insul. Mag..

[8] Li Y., Han J., Du Y., Jin H. (2023). Time-frequency maps for multiple partial discharge sources separation in cable terminations. IEEE Trans. Power Deliv..

[9] Gouda O.E., ElFarskoury A.A., Elsinnary A.R., Farag A.A. (2018). Investigating the effect of cavity size within medium-voltage power cable on partial discharge behaviour. IET Gener. Transm. Distrib..

[10] Qin C., Zhu X., Zhu P., Lin W., Liu L., Che C., Liang H., Hua H. (2024). Partial Discharge Signal Pattern Recognition of Composite Insulation Defects in Cross-Linked Polyethylene Cables. Sensors.

[11] Liu H., Li H., He N., Teng J., Cao B., Miao W., Bai R., Li X., Gao C. (2025). Design of an Ultra-High-Frequency Through-Core Current Transformer for Cable Partial Discharge Detection. Electronics.

[12] Kreuger F., Wezelenburg M., Wiemer A., Sonneveld W. (2002). Partial discharge. XVIII. Errors in the location of partial discharges in high voltage solid dielectric cables. IEEE Electr. Insul. Mag..

[13] Gao S., Liu H., Fan H. (2016). PD location method of power cable based on wavelet transform modulus maxima considering wave characteristics. Power Syst. Technol..

[14] Shu E.W., Boggs S. (2008). Dispersion and PD detection in shielded power cable. IEEE Electr. Insul. Mag..

[15] Ju T., Jiao C., Xiaoxing Z. (2009). Time difference algorithm based on energy relevant search of multi-sample applied in PD location. Proc. CSEE.

[16] Wagenaars P., Wouters P., Van Der Wielen P., Steennis E. (2008). Accurate estimation of the time-of-arrival of partial discharge pulses in cable systems in service. IEEE Trans. Dielectr. Electr. Insul..

[17] Liu J., Tang J., Lang Y. (2018). Approach for timedelay location of UHF signals of GIS partial discharge based on interpolating cross-correlation function. Gaoya Dianqi/High Volt. Appar..

[18] Liu W., Liu S., Hu X., Wei M., Zhang Y., Fan G. (2017). Aperiodic weak partial discharge source detection based on accumulation of cross correlation estimation information. High Volt. Eng.

[19] Kai Z., Xianjie R., Xianjin W. (2021). Application of distance based cross-correlation algorithm in partial discharge location of power cables. High Volt. Technol..

[20] Min X., Kai Z., Shilin Z. (2018). Research on partial discharge location using modified crosscorrelation method considering frequency characteristic of phase velocity. Power Syst. Technol..

[21] Mardiana R., Su C.Q. (2010). Partial discharge location in power cables using a phase difference method. IEEE Trans. Dielectr. Electr. Insul..

[22] Huangxi C., Chunhua F., Ziheng P. (2022). Double-ended positioning of partial discharge for long cable based on VMD-WVD phase method. Electr. Power Eng. Technol..

[23] Ghorat M., Gharehpetian G.B., Latifi H., Hejazi M.A. (2018). A new partial discharge signal denoising algorithm based on adaptive dual-tree complex wavelet transform. IEEE Trans. Instrum. Meas..

[24] Govindarajan S., Subbaiah J., Cavallini A., Krithivasan K., Jayakumar J. (2019). Partial discharge random noise removal using Hankel matrix-based fast singular value decomposition. IEEE Trans. Instrum. Meas..

[25] Wang X., Wang X., Gao J., Tian Y., Kang Q., Zhang F., Liu W. (2023). A denoising method for cable partial discharge signals based on image information entropy and multivariate variational mode decomposition. IEEE Trans. Instrum. Meas..

[26] Tang J., Zhou S., Pan C. (2019). A denoising algorithm for partial discharge measurement based on the combination of wavelet threshold and total variation theory. IEEE Trans. Instrum. Meas..

